# Titration of high dose sedation is effective in severe tetanus: a case report

**DOI:** 10.4076/1757-1626-2-6865

**Published:** 2009-09-09

**Authors:** Pan Chun, Huang Ying-Zi, Yang Yi, Qiu Hai-Bo

**Affiliations:** Department of Critical Care Medicine, Zhong-Da Hospital, Southeast University Clinical Medical SchoolNanjing 210009, Jiangsu ProvinceChina

## Abstract

**Introduction:**

Tetanus is a fatal infectious disease. It could cause typical signs like pain, headache, stiffness, and spasms of facial muscles as well as trunk and skeletal muscles. The symptoms are risus sardonicus, trismus and opisthotonus. How to control the spasticity and rigidity of muscles is still a problem. Our object is to raise the feasibility of titration of high dose sedatives in the management of severe tetanus.

**Case presentation:**

A 37-year-old woman was sustained a 2 cm wound in the right anterior part of chest. Then she developed progressive risus sardonicus, trismus and opisthotonus, elevated liver enzymes, creatine kinase, lactic acid and myoglobin. The patient was treated with continuous infusion of propofol (50-100 mg/h, 22 days) and midazolam (5-20 mg/h, 37 days) for sedation, vecuronium (1-6 mg/h, 25 days) for muscle relaxation. The symptoms of tetanus were controlled, and there were no side-effects appeared.

**Conclusion:**

We report one case of severe tetanus. In this case, several types of sedative were administrated and most of them were high doses. The patient recovered while no complications remained. This case report indicated that combination and high dose of sedation for severe tetanus were feasible. We recommend this treatment as the guidance of similar patients.

## Introduction

Tetanus is an acute fatal infectious disease which is caused by *Clostridium tetani*. The source of the infection is the contaminated wound, which often remains unidentified. Almost 1 million patients die of tetanus every year [1].

*Clostridium tetani* produces tetanus neurotoxin in anaerobic surroundings. Spreading to the central nervous system, tetanus neurotoxin prevents the release of the inhibitory neurotransmitter γ-amino butyric acid and then causes these typical symptoms of the disease [2]. Spasms are provoked by minor stimuli like sounds or touching, so physical examinations are difficult to perform. It has been described autonomic instability leads to fever, hypertension, tachycardia, and hypersalivation. Early onset of symptoms correlates with more severe disease. The diagnosis is entirely based on the clinical manifestations, while additional examinations are mainly done to exclude other diseases. Wound cultures are positive for *C. tetani* in only 30% of documented cases [3]. To prevent further neurotoxins absorption by surgical treatments and neutralizing neurotoxins is the aim of the treatment. For eradicating *C. tetani*, both penicillin and metronidazole are good choices. But high dose of penicillin in animal models was suggested increasing risk of convulsion, while metronidazole reduces the mortality and the stay in hospital [4]. Tetanus is a self-limiting disease. In approximately 2 to 4 weeks, the tetanus neurotoxins will be gradually cleared and the spasms will subside, but autonomic dysfunction will be present for several weeks [1].

Sedative is given in order to control the symptoms of tetanus, but there was no report in the guidelines for the doses of sedatives. In 1988, Helsinki University Central Hospital treated a severe tetanus adult patient with long-term infusions of propofol (20-80 mg/h, 11 days) and midazolam (5-15 mg/h, 29 days) for sedation, as well as vecuronium infusion (6-8 mg/h, 35 days) for muscle relaxation, and the patient recovered at last [5]. In this article, we treated our case of severe tetanus which was given combination and high doses of sedatives.

## Case presentation

Injured by firecracker on May 14th, 2008, a 37-year-old woman of Chinese Han individual sustained a 2 cm wound in the right anterior part of chest. She did not care about it and did not tell anybody because she felt ashamed. Then neck stiffness and muscle cramp in all extremities appeared gradually. Her husband sent her to the infectious hospital at first. The wound was cleaned and opened. She had been given tetanus antitoxin (1500 u) and a bolus of tetanus immune globulin (150 u/kg) in the following three days, but the symptoms developed with severe myoclonus, autonomic dysfunction and apnea. Diazepam (10 mg/30 min) was executed several times, but severe muscle spasms were still uncontrolled. The patient was in a coma, and the blood oxygen saturation was only 85%, then she was transferred to intensive care unit (ICU).

**Figure 1.**
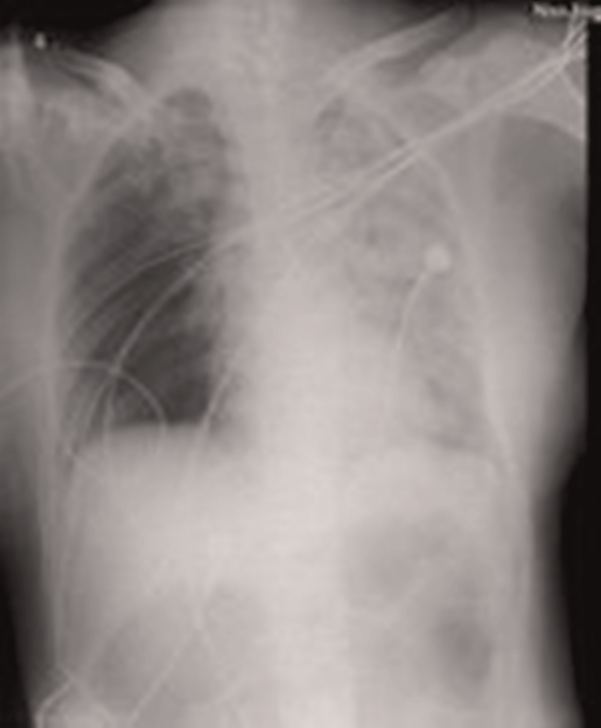
Chest X-ray film (the patient on admission of ICU).The left lung was severe compared to the right lung.

On admission of ICU, tracheal cannula and ventilator were given to help her breath. Risus sardonicus, trismus and opisthotonus could be observed. In a coma her heart rate was 171 per minute, blood pressure was 117/63 mmHg and SpO_2_ fell to 60%. Edema of bulbar conjunctiva was examined. With low respiratory sounds, crackles were heard, and the chest X-ray shows pneumonia of the left lung (Figure 1). Autonomic dysfunction with repetitive periods of tachycardia and profuse sweating developed. The elevated white blood cell count was 26.2 × 109/L, AST was 1454 u/L, LDH was 1127 u/L, CK was 23750 u/L, CK-MB was 163 u/L, pH 7.267 and the elevated lactic acid was 6.5 mmol/L. The diagnosis of the patient was severe tetanus, pneumonia, respiratory failure, myocardial damage. Propofol (300 mg/h), midazolam (5 mg/h), lytic cocktail (chlorpromazine 50 mg, pethidine 100 mg and promethazine 50 mg were dissolved in 50 ml physiological saline, 2 ml/h), morphine (2 mg/h) were intravenous administration continuously, and morphine (2-5 mg), diazepam (5-10 mg), phenobarbital (0.1 g) were used intravenously intermittently, but spasms and rigidity couldn’t be controlled. Then vecuronium bromide (8 mg/h) was administrated. Antibiotic, combination of glucose-insulin-potassium (GIK) were used to treat pneumonia and protect the cardiac. Sodium bicarbonate was also given to obviate acute renal failure which was caused by myoglobin.

Considering she needed ventilation a long time, we gave her tracheostomy the next day. To avoid the complications of tetanus, bronchofibroscope, prone ventilation and lung recruitment were performed, the concentration of myoglobin, ALT, CK and CK-MB were still elevated, and the triglyceride was not elevated obviously in the first week.

Although the patient was sedative deeply and the Ramsay scale was 6, the symptoms had not been controlled very well. Once the sedatives were stopped, spasms and rigidity would occur again, so we had to keep the patient sedated and observe her physical signs. However, the variability of blood pressure and heart rate was provoked by minor stimuli vigorously. Therefore she was given a bolus of propofol (30 mg) before any therapies.

After the second week in ICU, the hepatic function, the concentration of myoglobin, CK and CK-MB began to descend, but the triglyceride was elevated slightly. Propofol (50-100 mg/h), midazolam (20 mg/h), vecuronium bromide (4-6 mg/h) and morphine (2-4 mg/h) were infused continuously according to the reaction of the patient.

**Figure 2.**
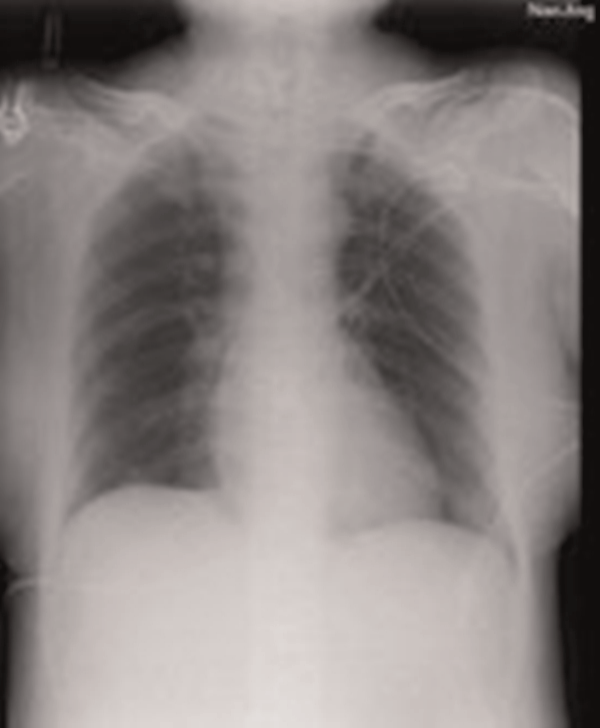
Chest X-ray film (25 days after on admission of ICU). Pneumonia was cured.

On 25th day in ICU, the pneumonia was controlled (Figure 2), spasticity and rigidity subsided, and no severe complications occurred, but sedatives were still needed [propofol (50 mg/h), midazolam (20 mg/h), vecuronium bromide (4 mg/h)]. In this condition, we decided to withdraw the sedatives gradually. At first midazolam was decreased to a rate of 10 mg/h, but diaphoresis occurred, and her heart rate increased to 150 per minute, BP increased to 180/100 mmHg. Luckily, there were no spasms. Considering the withdrawal syndrome, midazolam was increased to 20 mg/h. Then the symptoms disappeared. The vecuronium bromide infusions were stopped firstly on a regular basis to ensure optimal sedation levels and monitor the progression of disease. While monitoring the symptoms, fluctuations of blood pressure and heart rate, the dosage of propofol and midazolam were reduced gradually. Spasms subsided gradually both in frequency and severity which allowed the reduction of their infusion rates.

After 5 weeks of intensive care, the patient awakened. We stopped all of the sedatives gradually, except midazolam (2-4 mg/h). However, because of lying in bed for nearly one month her limbs couldn’t move and sometimes trembled. After stoping using midazolam on the 45th day, we decided to help the patient to wean from ventilator. On the 52th day in ICU, she weaned successfully, the concentration of myoglobin, hepatic function, CK and CK-MB were normal, and the elevated triglyceride was 1.9 mmol/L. On 57th day, she was decannulated, and with rehabilitation exercise she could stand and live as before. No complications were observed.

## Discussion

The germinated spore of tetanus bacillus secretes two toxins: tetanospasmin and tetanolysin. Tetanolysin can help the diffusion of tetanospasmin under anaerobic conditions in necrotic or infected tissues which surrounds the infection area. Tetanospasmin primarily affects inhibitory neurones, prevents the release of the neurotransmitters glycine and γ-amino butyric acid, and leads to failure of inhibition of motor reflex responses to sensory stimulation [1]. These are the causes for the muscle rigidity of a tetanic spasm. The worst hazard of tetanus is spasm especially the uncontrolled one. Spasms may cause laryngeal obstruction, a reduction in chest wall compliance, and respiratory failure which is the most common direct cause of death from tetanus [6]. Spasms also cause aspiration time and again, induce myoglobin to release, make blood pressure and heart rate fluctuate vigorously, and pneumonia, acute renal failure and heart failure occur. In order to prohibit the progression of the disease, three goals must be achieved. The most important one is the neutralization of any circulating toxin before spreading to the central nervous system and producing further effects. This is accomplished by intravenous and intramuscular administration of adequate doses of tetanus antitoxin as soon as possible. The second goal is to control infection to prevent further production of toxins. The last goal is to treat spasticity and rigidity. The first two goals may be achieved easily, but the third one is difficult [4].

A review article discussed the management of tetanus, which had traditionally been with sedation, anticonvulsants, neuromuscular blocking agents, and positive pressure ventilation [7]. For the patient with infection of tetanus, firstly the environment in which the tetanus lives should be extinct; secondly the toxins should be eliminated and neutralized; thirdly incision of trachea and pulmonary ventilation are needed to avoid infection; finally spasticity and rigidity should be controlled, otherwise the complication of tetanus will appear. In this case the patient got infected with tetanus then showed continuous spasms when she was sent to ICU. There was high level of myoglobin in blood, pneumonia and hepatic function injured. Fortunately, renal failure and heart failure did not occur.

It is necessary to give the patient enough sedation to control the spasticity and rigidity. The choice of sedatives is difficult sometimes. The sedatives which can block the toxins to the acceptors should be used. Diazepam compared to other drugs has the advantages of combined anticonvulsant, muscle relaxant, sedative and anxiolytic effects. In 2004 Okoromah CN did a randomized and quasi-randomized controlled trial, meta-analysis of in-hospital deaths indicated that children treated with diazepam alone had more chance of survival than those treated with combination of phenobarbitone and chlorpromazine [8]. Also in 2004, Ismoedijanto treated the children with severe tetanus using high dose diazepam, he suggested that the dose of diazepam exceeds 240 mg per day to a child, and a ventilator should be on hand and if the dose required is more than 480 mg per day, other drugs should be considered [9]. But single sedative can’t be efficient in controlling the spasticity and rigidity for adult. Drug combination is the only way to control the symptoms. Benzodiazepines, propofol, morphine, muscle relaxant and other sedatives should be considered.

Some other medicines are also used for controlling the symptoms of tetanus. In 2005, Beecroft used remifentanil infusion which was started at a rate of 0.05-0.1 mg/kg.min as an adjunct to sedation and for analgesia, and the infusion rate was increased 1 mg before any therapy, and titrated to effect (0.3-0.6 mg/kg.min) to control spasms very well and withdraw the other medicines gradually [10]. In 2006, Dr CL Thwaites did a randomised, double blind placebo controlled trial in 256 Vietnamese patients. Participants were randomly assigned magnesium sulphate (n = 97) or placebo solution (n = 98) intravenously for 7 days. The result showed that magnesium infusion did not reduce the need for mechanical ventilation in adults with severe tetanus but reduced the requirement for other drugs to control muscle spasms and cardiovascular instability [11].

But what are the appropriate doses of sedatives? Do high doses of sedatives bring the side-effect to the patients? In specifications of drugs, high doses of propofol could bring hyperlipidemia and propofol infusion syndrome, while high doses of vecuronium bromide could induce neuromuscular blockade and respiratory dysfunction. There are no instructions guiding doctors to give the proper doses to this kind of patients. In 1988, Helsinki University Central Hospital treated an adult patient of severe tetanus with long-term infusions of propofol (20-80 mg/h, 11 days) and midazolam (5-15 mg/h, 29 days) for sedation, and with vecuronium infusion (6-8 mg/h, 35 days) for muscle relaxation. The patient recovered, but he was unable to recall anything about his stay in the ICU [5]. The patient in our ICU was treated with long-term infusions of propofol (50-100 mg/h, 22 days) and midazolam (5-20 mg/h, 37 days) for sedation, and with vecuronium infusion (1-6 mg/h, 25 days) for muscle relaxation. The patient was not found tolerance to propofol, but when we decided to withdraw midazolam, withdrawal syndrome occurred, we thought she develop tolerance to midazolam. Propofol infusion syndrome also was not found, cardiac function and hepatic function were not aggravation with the recovery of tetanus. But the triglyceride was elevated slightly.

It’s difficult to assess the degree of sedation. According to the guideline of sedation, the sedation goal or endpoint should be established and redefined regularly. Regular assessment and patients’ response to therapy should be systematically documented [12]. But the degree of sedation of these patients can’t be judged by Ramsay scale as usual. Patients who cannot communicate with others should be assessed through subjective observation of pain-related behaviors (movement, facial expression, and posturing) and physiological indicators (heart rate, blood pressure, and respiratory rate) and changes of their physical parameters.

## Conclusion

We report one case of severe tetanus. In this case several sedatives were administrated and most of them were high doses. The patient recovered while no complications remained. For spasticity and rigidity induced by severe tetanus, combinations and high doses of sedatives should be considered and subjective observation, physiological indicators and changes of their physical parameters can be used to access the degrees of sedation. The proposal of combining overdosage of several sedatives is available in treatment of patients with this kind of symptom.

## Abbreviations

AST: aspartate aminotransferase
CK: creatine kinase
CK-MB: MB isoenzyme of creatine kinase
*C. tetani*: *Clostridium tetani*
ICU: intensive care unit, LDH, lactate dehydrogenase
SpO_2_: blood oxygen saturation
